# Endovascular and Open Surgical Treatment of Ruptured Splenic Artery Aneurysms: A Case Report and a Systematic Literature Review

**DOI:** 10.3390/jcm12186085

**Published:** 2023-09-20

**Authors:** Luigi Federico Rinaldi, Chiara Brioschi, Enrico Maria Marone

**Affiliations:** 1 Vascular Surgery, Department of Integrated Surgical and Diagnostic Sciences, University of Genoa, 16132 Genoa, Italy; 2 Vascular Surgery, Ospedale Policlinico di Monza, 20900 Monza, Italy; chiara.brioschi@policlinicodimonza.it (C.B.); enricomaria.marone@gmail.com (E.M.M.); 3 Vascular Surgery, Department of Clinical-Surgical, Diagnostic and Pediatric Sciences, University of Pavia, 27100 Pavia, Italy

**Keywords:** ruptured splenic aneurysms, splenic artery aneurysms and pseudoaneurysms, visceral aneurysms, splanchnic aneurysms, transarterial artery embolization, splenectomy, aneurysm rupture, shock during pregnancy

## Abstract

Background: Ruptured splenic artery aneurysms (r-SAA), although rare, are burdened by high morbidity and mortality, even despite emergent surgical repair. It is suggested that endovascular treatment can achieve reduction in peri-operative death and complication rates, as in other vascular diseases, but evidence of such benefits is still lacking in this particular setting. We report a case of an r-SAA treated by trans-arterial embolization and then converted to open surgery for persistent bleeding, and we provide a systematic review of current results of open and endovascular repair of r-SAAs. Materials and Methods: A 50-year-old male presenting in shock for a giant r-SAA underwent emergent coil embolization and recovered hemodynamic stability. On the following day, he underwent laparotomy for evacuation of the huge intraperitoneal hematoma, but residual bleeding was noted from the splenic artery, which was ligated after coil removal, and a splenectomy was performed. A systematic literature review of the reported mortality and complications of r-SAA undergoing open (OSR) or endovascular (EVT) treatment was performed using the main search databases. All primary examples of research published since 1990 were included regardless of sample size. The main outcome measures were mortality and reinterventions. Secondary outcomes were post-operative complications. Results: We selected 129 studies reporting on 350 patients—185 treated with OSR and 165 with EVT. Hemodynamically unstable patients and ruptures during pregnancy were more frequently treated with open repair. Overall, there were 37 deaths (mortality: 10.6%)—24 in the OSR group and 13 in the EVTr group (mortality: 12.9% and 7.8% respectively, *p*-value: 0.84). There were 37 reinterventions after failed or complicated endovascular repair —6 treated with endovascular re-embolization and 31 with laparotomy and splenectomy (22.4%); there were 3 (1.6%) reinterventions after open repair. Overall complication rates were 7.3% in the EVT group (*n*: 12) and 4.2% in the OSR group (*n*: 7), and did not require reintervention. No significant differences in overall complications or in any specific complication rate were observed between the two groups. Conclusions: Current results of r-SAA treatment show equipoise terms of morbidity and mortality between open and endovascular repair; however, in case of hemodynamic instability and rupture during pregnancy, open surgery might still be safer. Moreover, endovascular repair is still burdened by a significantly higher rate of reinterventions, mostly with conversions to open surgery.

## 1. Introduction

Endovascular repair is imposing itself as the first-choice elective treatment of visceral artery aneurysms (VAA) and pseudoaneurysms (VAPA), when anatomically feasible. However, in urgent and emergent settings, especially in cases of hemodynamic instability, most guidelines and current literature still recommend laparotomy and open surgical repair, depending on the aneurysm location [1,2]. The treatment of ruptured aneurysms of the splenic artery (SAA), in particular, is among the most controversial issues, in the absence of clear evidence favoring open or endovascular repair [3].

SAAs are the most common visceral artery aneurysm (60% of all VAAs) and the third most common abdominal aneurysm [1,2,3]. Risk factors for a true SAA or pseudoaneurysm (SAPA) are portal hypertension, liver transplantation, pancreatitis, segmental mediolysis, and other forms of vasculitides. It is more frequent in women, and the risk of rupture is high during pregnancy, with extremely high mortality and morbidity for both mothers and fetus [1,3]. In case of rupture, the prognosis is poor, even in case of prompt open or endovascular treatment [1].

The aim of this study is to report a case of ruptured splenic artery aneurysm (r-SAA) treated by embolization and converted to open repair on post-operative day I, and to revise the current literature reporting on the mortality, complications, and conversion rates of open and endovascular treatment of such diseases.

## 2. Materials and Methods

### 2.1. Case Report

**Figure 1:**
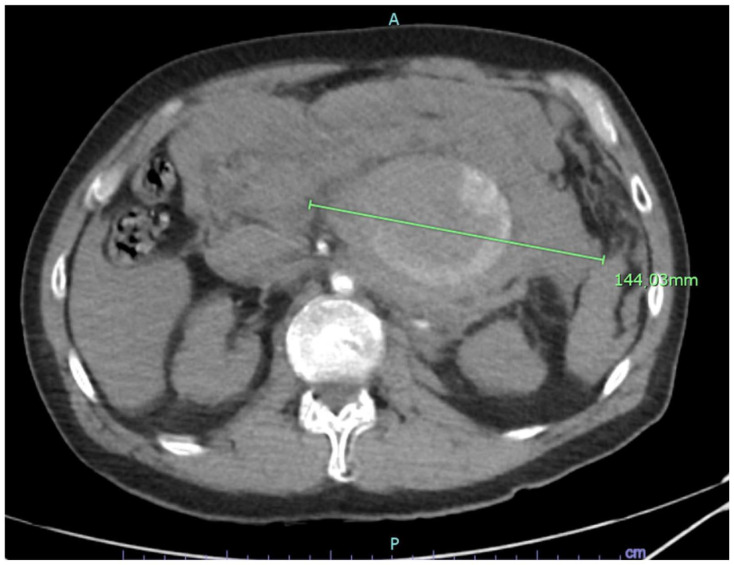
Axial view of a huge, ruptured SAA. A: anterior; P: posterior.

**Figure 2:**
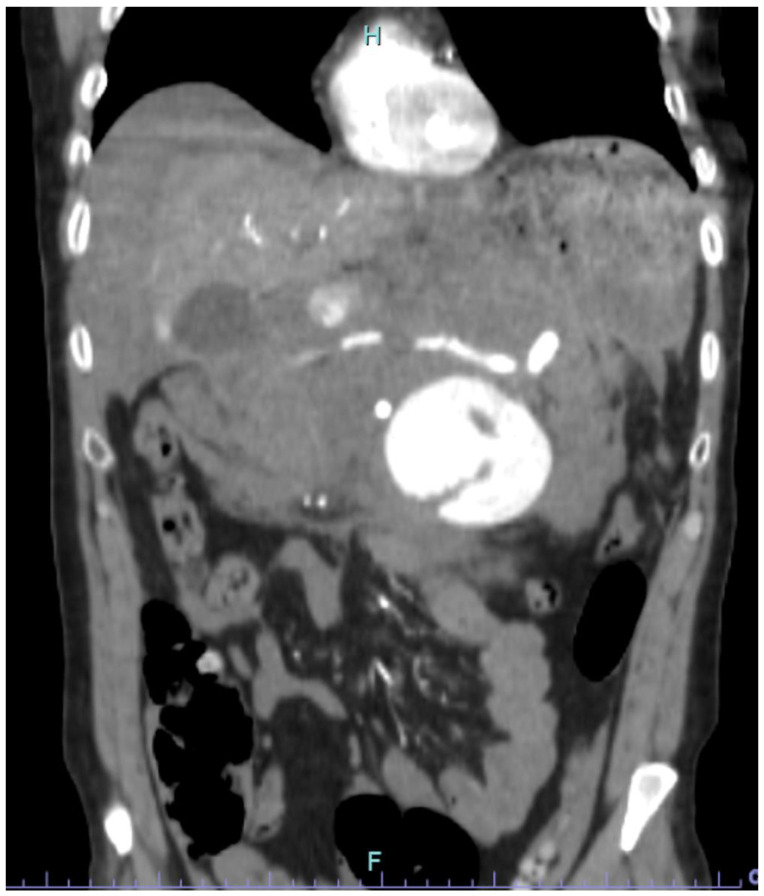
Ruptured SAA in coronal view.

A 50-year-old male with no previous cardiovascular history presented to the Emergency Room of our Center with severe abdominal pain and hypotension, which soon evolved into hemorrhagic shock with loss of consciousness. He was an active smoker, but past medical and surgical history were silent. Laboratory tests revealed severe anemia (HB: 7 g/dL) and severe impair of the coagulation (INR: 3, aPTT ratio: 2.2). He underwent emergent Computed Tomography Angiography (CTA), which revealed a giant intraperitoneal hematoma arising from an r-SAA with a maximum diameter of 10 cm (Figure 1 and Figure 2).

**Figure 3:**
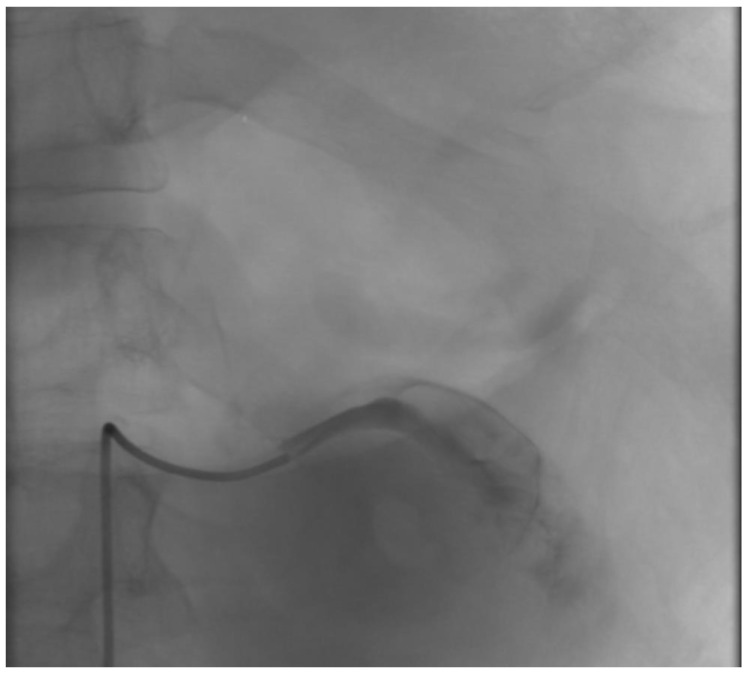
Selective intraoperative angiography showing a ruptured SAA.

**Figure 4:**
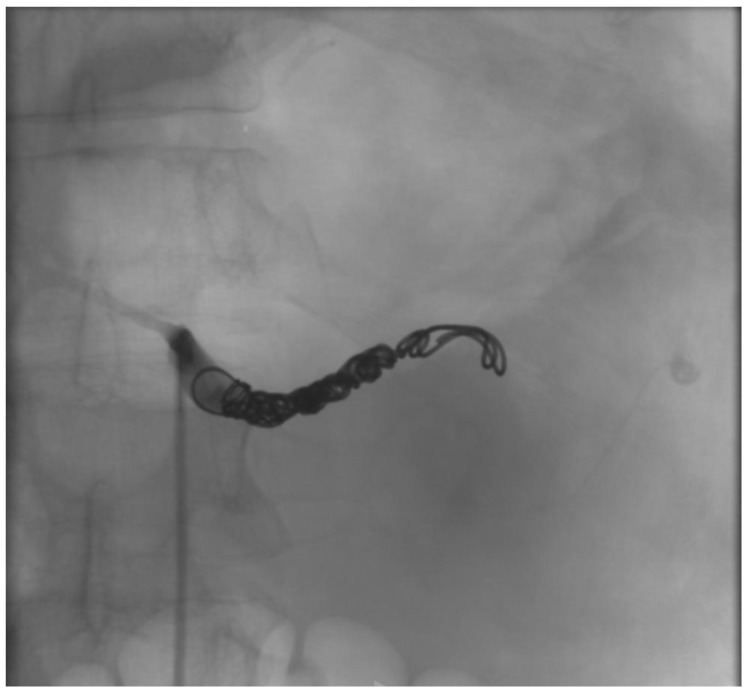
Proximal coil embolization of the splenic artery.

**Figure 5:**
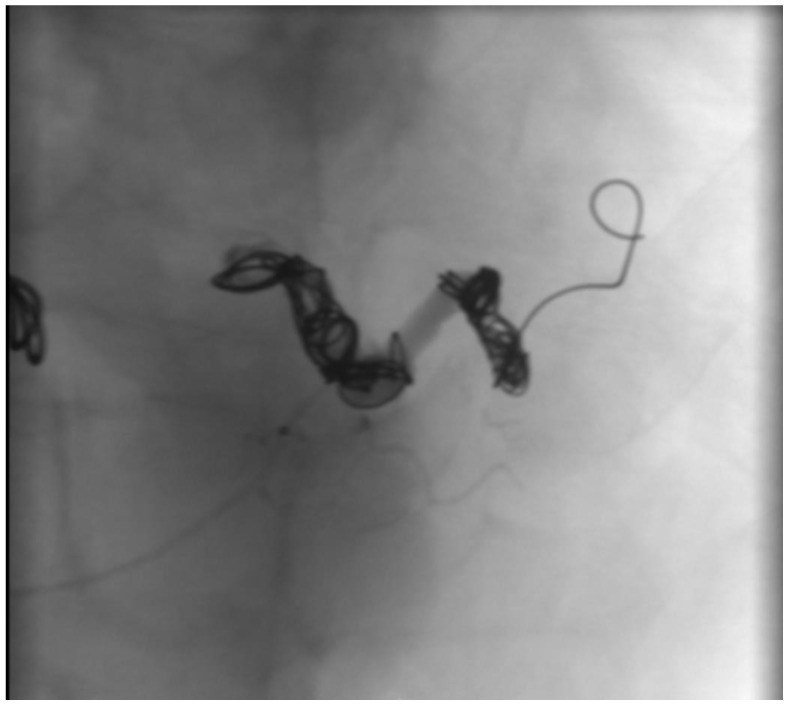
Proximal and distal embolization of the SA.

**Figure 6:**
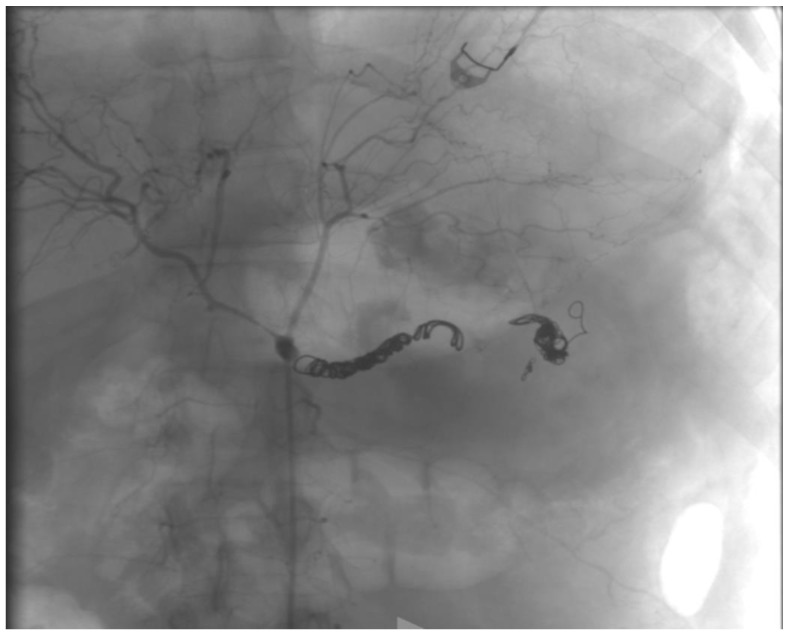
Final angiogram showing successful aneurysm exclusion.

The patient presented with a severe alteration of all the coagulation parameters, and the CTA showed a huge aneurysm involving the most distal portion of the splenic artery; we thought that outright laparotomy and splenectomy would have made the hemorrhage worse. Moreover, the vascular control of the splenic aneurysm was very difficult to obtain, due to the aneurysm size and the difficulty to isolate the splenic artery. For those reasons, after a multidisciplinary evaluation involving radiologists and general and vascular surgeons, we preferred an endovascular approach, and the patient underwent emergent angiography under general anesthesia. The coeliac artery was engaged through a 6F left femoral access, and after selective angiography confirmed rupture of SA, proximal embolization with 6 detachable metallic coils (Interlock-18^®^ and Interlock-35^®^, Boston Scientific, Marlborough, MA, USA), sized 8, 10, and 12 mm, was performed. The angiography showed residual bleeding from a pancreatic branch that joined the distal splenic artery in the most lateral portion of the aneurysm, so we engaged the pancreatic artery through superselective catheterization with a microcatheter and released 3 metallic coils (Interlock-18^®^, Boston Scientific, Marlborough, MA, USA), sized 8 mm, in the distal splenic artery and in the pancreatic artery right above its anastomosis with the splenic artery (Figure 3, Figure 4 and Figure 5). The hemorrhage was effectively controlled and final completion angiogram from the coeliac trunk showed no signs of contrast extravasation (Figure 6). The patient regained hemodynamic stability, but, due to his severely altered coagulative parameters, the prospected laparotomy to drain the intraperitoneal hematoma and prevent abdominal compartment syndrome was postponed to the following day. Meanwhile, the patient received red blood cell, fluid, and plasma integration, and remained intubated and stable throughout the night.

**Figure 7:**
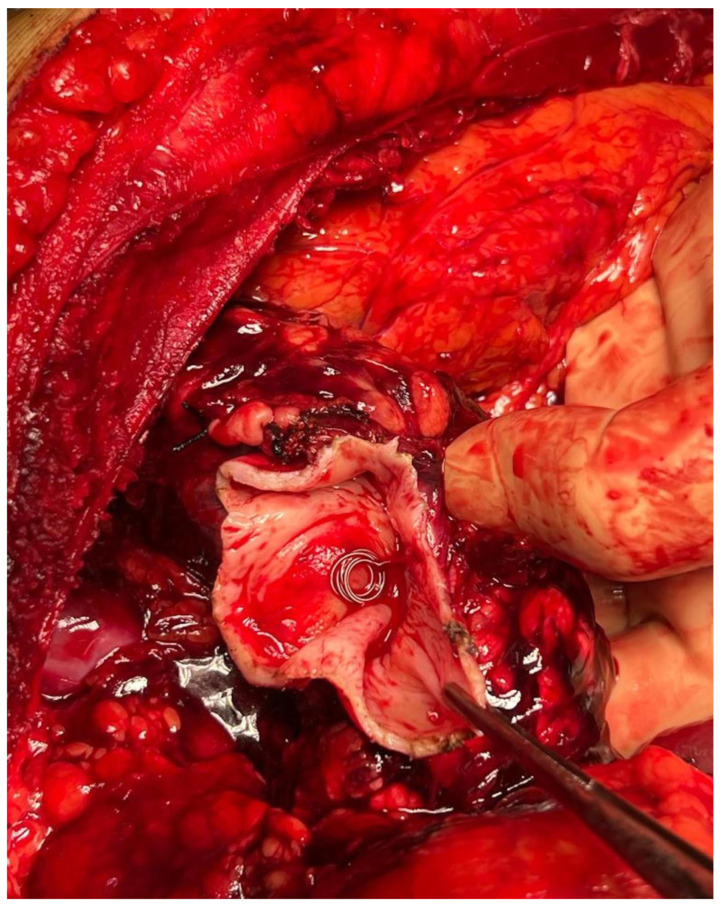
Coil removal after laparotomy.

**Figure 8:**
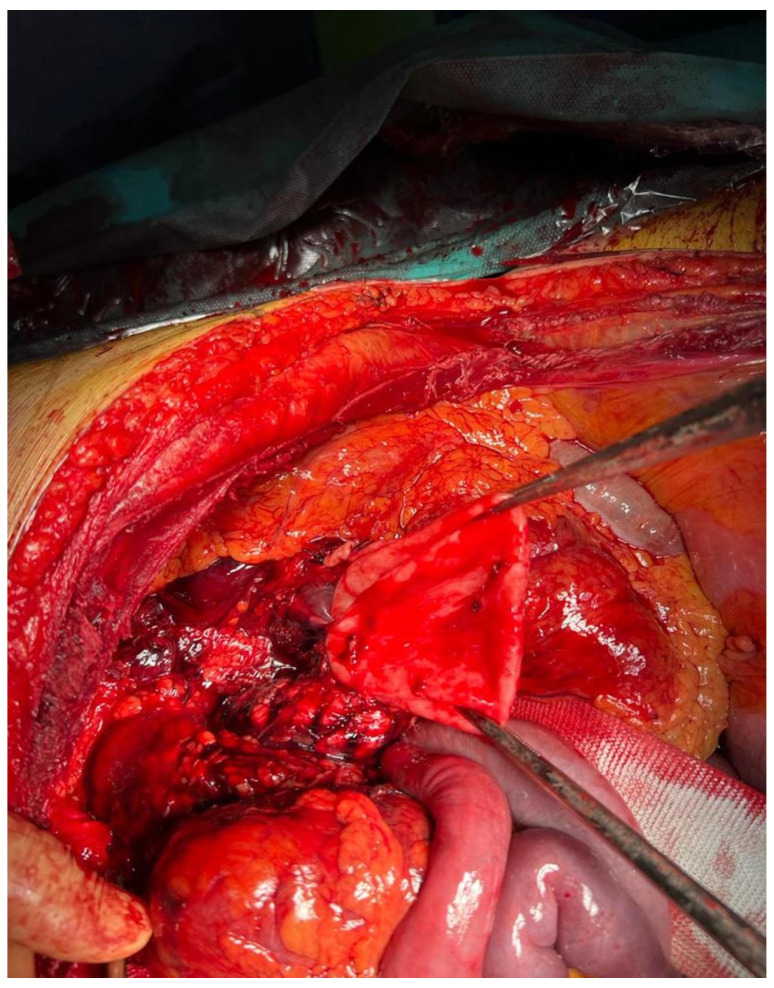
SAA excision.

On the following day, a chevron laparotomy was performed, but while opening the lesser omentum, massive bleeding was observed that required supracoeliac aortic clamping. We isolated the coeliac trunk and the giant splenic aneurysm and ligated its arterial inflow and outflow. We drained the hematoma, removed the coils placed into the afferent and efferent vessels, and resected the aneurysm along with the spleen (Figure 7 and Figure 8). After that, the hemorrhage was fully controlled, and the surgical incision was closed after leaving a drain in the splenic lodge. The patient left the operative room stable, but he deceased a few hours after surgery due to multiorgan failure and severe hyperkaliemia.

### 2.2. Literature Review

#### 2.2.1. Search Strategy

Cochrane, Embase, and PMC databases were searched using the following search strategy:

“(((visceral OR splanchnic OR splenic) AND ((aneurysm [Title/Abstract]) OR (pseudoaneurysm [Title/Abstract])) AND ((rupture [Title/Abstract]) OR (ruptured visceral aneurysm [Title/Abstract]))) NOT (((aortic [Title/Abstract]) OR (abdominal aortic [Title/Abstract]) OR (thoracoabdominal [Title/Abstract])))”. The last search was made on 1 August 2023.

**Figure 9:**
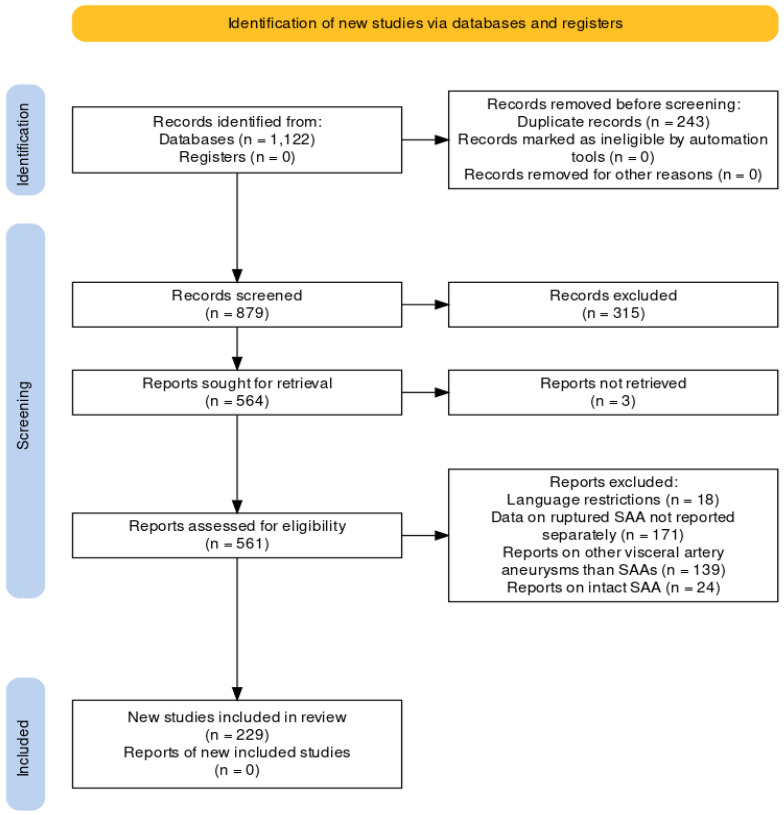
Selection flow-chart of the reports included in the review.

Study selection and analysis was conducted in accordance with the Preferred Reporting Items for Systematic Reviews and Meta-Analysis (PRISMA) Checklist (Figure 9) [4]. This systematic review was not registered on platforms.

#### 2.2.2. Inclusion and Exclusion Criteria

We included all comparative and non-comparative studies published since 1990 in English, French, German, Spanish, or Italian reporting on open or endovascular repair of ruptured splenic artery aneurysms (r-SAAs) and pseudoaneurysms (r-SAPAs). Studies published in other languages were included only if they had an abstract in English providing all the useful data. All primary sources of research were included regardless of sample size, as long as they reported on mortality, morbidity, and rates of reintervention and open conversion. Studies not reporting on aneurysm location, treatment technique, and peri-operative and post-operative results were not included. Studies reporting on both intact and ruptured visceral aneurysms were included only if separated results for the two groups were provided. The results were screened manually; study selection and inclusion and exclusion criteria were assessed independently by 2 authors (LFR and CB); in case of contrasting results, a third author decided (EMM).

#### 2.2.3. Outcome Measures

The endpoints were mortality and reintervention/conversion rates of endovascular and open repair. Mortality was defined as death by any cause reported within 30 days since the intervention, and any surgical-related or aneurysm-related death reported afterwards.

### 2.3. Statistical Analysis

Categorical variables were expressed as percentage, continuous variables as mean and standard deviation. Chi-square and t-test were employed to compare the distribution of nominal and ordinal variables respectively between the two groups. Odds ratios of mortality and reinterventions rates were calculated for patients treated by open and endovascular repair and two-sided *p*-values < 0.05 were considered significant. Logistic regression analysis was performed to ascertain if hemodynamic instability was a predictor of mortality or reintervention.

## 3. Results

### 3.1. Study Selection

We identified 1122 records. After examining inclusion and exclusion criteria and removing duplicates, we selected 227 studies reporting on 350 patients. Most studies (197) were case reports, 29 were series reporting on >2 cases and 3 were comparative retrospective studies.

### 3.2. Findings

#### 3.2.1. Patients

Overall, 120 males, 95 females, and 135 patients whose gender was not reported received open surgical repair (OSR) or endovascular treatment (EVT) for an r-SAA (258) or a r-SAPA (92) [5,6,7,8,9,10,11,12,13,14,15,16,17,18,19,20,21,22,23,24,25,26,27,28,29,30,31,32,33,34,35,36,37,38,39,40,41,42,43,44,45,46,47,48,49,50,51,52,53,54,55,56,57,58,59,60,61,62,63,64,65,66,67,68,69,70,71,72,73,74,75,76,77,78,79,80,81,82,83,84,85,86,87,88,89,90,91,92,93,94,95,96,97,98,99,100,101,102,103,104,105,106,107,108,109,110,111,112,113,114,115,116,117,118,119,120,121,122,123,124,125,126,127,128,129,130,131,132,133,134,135,136,137,138,139,140,141,142,143,144,145,146,147,148,149,150,151,152,153,154,155,156,157,158,159,160,161,162,163,164,165,166,167,168,169,170,171,172,173,174,175,176,177,178,179,180,181,182,183,184,185,186,187,188,189,190,191,192,193,194,195,196,197,198,199,200,201,202,203,204,205,206,207,208,209,210,211,212,213,214,215,216,217,218,219,220,221,222,223,224,225,226,227,228,229,230,231]. Mean age was 47.2 ± 17 years. We found an extreme variability among the reported r-SAA diameters, ranging between 20 mm (mainly PSA) to giant SAAs measuring 14 cm. The mean diameter was 53 mm, with a standard deviation of 21.

Overall, 185 patients received open surgery with ligation and aneurysm excision; splenectomy was performed in 179 cases (96.7%); a distal pancreasectomy and left hemicolectomy were necessary to complete the vascular control in 8 and 4 cases, respectively.

Overall, 165 patients underwent endovascular repair. Most endovascular treatments (160/165) were conducted through transarterial embolization (TAE), mainly with metallic coils or microcoils, liquid agents (12), or a mixture of the two with or without thrombin injection (15) and in one case with an Amplatzer plug. Covered stents were used in three cases, and in two cases the technique was not specified.

Distribution of age, sex, and clinical characteristics between the two groups are reported in Table 1. Patients who had undergone open repair were significantly younger and more likely to be female, to have an ongoing pregnancy, and to present with hemodynamic instability—defined as shock or hypotension at presentation. Pseudoaneurysms were more frequent in patients treated by endovascular means.

#### 3.2.2. Outcomes

Overall, there were 37 deaths (mortality: 10.6%), 35 of them within the first 30 days after treatment (Table 2). The leading causes of mortality were disseminated intravascular coagulation (DIC) in 9 cases, multiorgan failure (MOF) in 7 cases, sepsis in 5 cases, ischemic colitis in 3 cases, cardiac complications in 2 cases, and respiratory distress syndrome in 1 case. In 9 cases, the causes of death were not clearly reported. Overall complication rates were 7.3% in the EVT group (12 patients) and 4.2% in the OSR group (7 patients): in 8 cases, they were systemic (2 sepsis, 2 myocardial infarctions, 1 stroke, 1 respiratory failure, and 2 bowel obstructions, Table 3). Local complications occurred in 7 cases in the EVT group (3 splenic abscesses and 4 splenic infarction) and in 3 cases in the OSR group (1 splenic abscess and 2 pancreatitis) and did not require reintervention. No significant differences in overall complications or in any specific complication rate were observed between the two groups.

Based on intention-to-treat analysis, the reported mortality rate was 12.9% in OSR and 7.8% in EVT groups, *p*-value: 0.84. The reintervention rate after failed or complicated endovascular repair was 22.4% (37 cases, 6 treated with endovascular re-embolization and 31 with laparotomy and splenectomy) (Table 2). The leading causes for open conversion were recurrent or residual bleeding (26 cases), failure to selectively catheterize the splenic artery (5 cases), and the need to drain an intraperitoneal hematoma to prevent abdominal compartment syndrome (6 cases). Reintervention after open repaired was reported in 5 cases (2.7%), all for hemostasis completion.

Overall, 86 cases of r-SAAs concerned pregnant women, 7 of whom underwent TAE and 79 OSR. The preference for OSR in pregnancy is probably due to the radiation exposure of fetuses for pregnant patients treated with EVT.

Overall maternal and fetal mortality was 16.3% and 32.5%, respectively (14 maternal and 28 fetal deaths). Mortality rates were similar between endovascular and open repair groups: in the first one, 1 maternal and 2 fetal deaths were reported (14.3% and 26.6%, respectively); in the latter, maternal and fetal mortality were 16.5% and 33%, respectively (N: 13 and 26).

#### 3.2.3. Regression

Logistic regression analysis confirmed hemodynamic instability as a predictor of mortality in patients undergoing OSR and a predictor of reintervention in patients undergoing EVT (Table 2).

## 4. Discussion

This systematic review found no significant differences in mortality rates between endovascular and open treatment of ruptured splenic artery aneurysms and pseudoaneurysms, but our results confirm that endovascular repair carries higher reintervention and conversion rates than open surgery, as observed in many other vascular diseases, especially in case of hemodynamically unstable patients.

The issue of treatment choice of ruptured visceral vessels is currently object of debate. In fact, although endovascular treatment, especially TAE, has now become the first-line treatment option for ruptured visceral aneurysms and pseudoaneurysms, especially those of the pancreatic arcade and of the distal branches of the mesenteric arteries. The Guidelines are still extremely cautious in recommendations when it comes to ruptured splenic aneurysms.

The latest Guidelines issued by the Society for Vascular Surgery, for example, recommend ligation with or without splenectomy of r-SAAs discovered on laparotomy and open or endovascular treatment of r-SAA diagnosed on previous imaging, based on the patient’s anatomy and baseline clinical conditions [1].

This is based on the most recent Metanalysis by Barrinuevo et al., which reported high mortality for open repair of r-SAAs (0.29) but provided no data on their endovascular repair [232]. Moreover, that study included an extremely heterogeneous corpus of case series, most of which did not provide separated outcomes for ruptured and non-ruptured SAAs. This makes it extremely difficult to estimate the actual mortality and morbidity rates related to the two treatment options.

**Figure 10:**
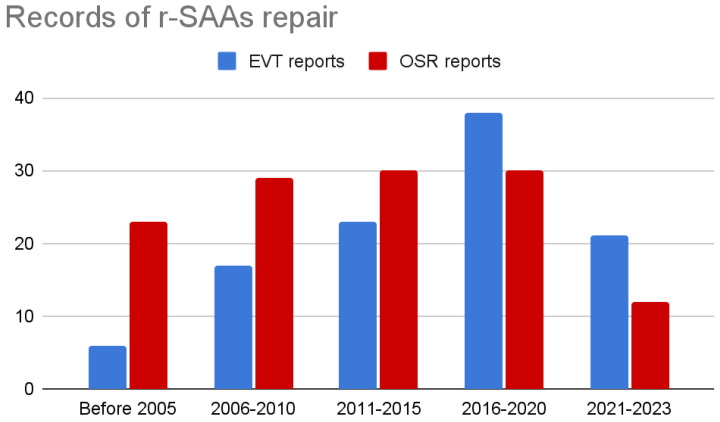
Records of r-SAA repair between 1990 and 2023.

It is known that peri-operative mortality of r-SAAs and r-SAPAs is extremely high, ranging between 20% and 30%, with a considerable risk of fetal and maternal death if rupture occurs during pregnancy [3]. For this reason, urgent endovascular repair is considered a promising possibility to decrease intraoperative mortality, complications, and length of in-hospital stay, but the current data have failed to show better outcomes in the treatment of r-SAA and r-SAPA, and hemodynamic instability is still seen as a setting in which the open approach should be preferable [233,234]. On the other hand, there are many reports in the current literature that describe successful endovascular treatment of ruptured splenic arteries, and their number is growing in frequency in the latest years, as Figure 10 indicates. The uncertainty on this subject is also caused by the rare occurrence of SAAs presenting with rupture and the heterogeneity in outcomes of specific studies reporting on the post-operative outcomes of their urgent repair, which is the goal of this review.

Revising all available reports on r-SAAs and r-SAPAs treated by open or endovascular surgery in the last 30 years, we found a significantly lower overall mortality rate than previously reported (10.9%), which could be attributable to a publication bias concerning the many case reports included in the review [234]. Mortality rates did not differ significantly between the two groups, but endovascular treatment carries a significant higher risk of early conversion or reintervention, consistently with the data reporting on elective repair of SAAs [232].

Notably, we observed that the ratio between r-SAA treated by surgical-first vs. endovascular-first strategy is changing in favor of the latter, in the most recent years, especially when pseudoaneurysms and elderly patients are involved. TAE results by far the preferred technique, with a much lower incidence of ischemic complications than usually reported (4 splenic infarctions; 2.4% vs. 38% according to Cordova et al. [233]), and often without clinical significance. TAE is particularly useful in bleeding and ruptured aneurysms because it achieves faster and usually effective control of the hemorrhage, allowing the patient to recover his stability, and in case of failure it can be easily repeated. The true Achilles heel of TAE is early and late coil migration, which often require reintervention and open conversion, particularly in hemodynamically unstable patients, like in the case here reported.

Concerning open surgery, splenectomy, although it is not routinely recommended even in emergent r-SAA repair, is still widely employed in emergency surgery in order to optimize bleeding control and prevent infective complications, such as splenic abscesses [1,2]. The main concerns after splenectomy are sepsis from capsulated bacteria, spleno-mesenteric vein thrombosis, and pancreatitis, although the data collected in this regard did not show higher systemic or local complication rates as compared with EVT.

Hemodynamic instability at presentation was confirmed as an important predictor of perioperative death in OSR group, whereas the numbers were too small to prove the same correlation in the EVT group; hemodynamically unstable patients who had undergone endovascular interventions were, however, more prone to have reintervention or open conversions. Conversion could be due to incomplete aneurysm exclusion or abdominal compartment syndrome due to the intraperitoneal hematoma, as shown in our case report. Although the patient was stable after EVT, he still had severe coagulation impairment after the procedure, and we planned decompression laparotomy for the following day. Unfortunately, during this phase, the coils migrated, and the patient started to bleed again. If a conversion is planned to optimize hemostasis, it is better to perform it right after embolization, and that is a lesson learned for us that we would like to share with our readers.

Such observations help to shed a light on mortality and complications data: in fact, although the observed rates between OSR and EVT groups were similar, hemodynamic instability and rupture during pregnancy, which are known predictors of worse prognosis, were significantly more frequent among patients treated by open surgery, suggesting that OSR may be safer than EVT in such circumstances.

### Limitations

This review has several limitations. First, the heterogeneity of the data, which come mainly from case reports, case series, and portions of larger comparative studies conducted on VAAs, from which the data regarding r-SAAs and r-SAPAs were extracted. This also causes a wide heterogeneity in how results were reported and resulted in us excluding many studies in which the specific outcomes of r-SAAs and r-SAPA were not clearly provided [235,236,237]. In fact, the largest and most interesting studies on this topic report on VAAs considered as a whole, sometimes conducting subgroup analysis by aneurysm location or by symptomatic/asymptomatic presentation; visceral artery aneurysms should be seen instead as separated identities, each with its peculiarities, because of the differences in anatomy and physiology of the abdominal organs they supply. The latest SVS Guidelines, for the first time, endeavor to provide recommendations concerning the treatment choice based on aneurysm locations, but we still need more evidence to strengthen the value of their assessments, especially in the field of ruptured VAAs. To produce high-quality evidence in this respect, reviews and metanalysis should rely on better quality primary studies reporting separately the outcomes of each aneurysm type and distinguishing between intact and ruptured VAAs [238].

The second limitation concerns the sample size: although 350 r-SAAs and VAPAs are in theory a large number, the number of reported deaths and complications is lower than reported in other studies [233,234]. This may have biased the statistical analysis, especially the logistic regression trying to establish the role of hemodynamic status as a mortality predictor in the EVT group. A publication bias is probably responsible for such discrepancy in mortality and morbidity.

Another important limitation is that the compared patient groups present statistically significant difference in their baseline clinical characteristics, along with the heterogeneity of the reports, but this is a common problem of retrospective studies. On the other hand, the equipoise in mortality, although patients treated with EVT were younger and more likely to be hemodynamically stable, strengthens the conclusion that EVT is not superior to OSR in emergent setting.

Finally, the review is an attempt to provide new insights on the outcomes of emergent treatment of r-SAAs, focusing on mortality and reintervention, but there are other minor outcome measures that could be considered in a more detailed analysis, such as the length of post-operative recovery and in-hospital stay, the quality of life, and the treatment-related costs. Moreover, mid-term and longer-term outcomes, particularly late conversions, are also worthy of assessment. Unfortunately, such information is seldom reported in primary literature.

## 5. Conclusions

Rupture of a splenic artery aneurysm is burdened by high mortality and morbidity, and its optimal management is still controversial. Although the popularity of endovascular treatment is increasing even in an emergency setting, there is still no evidence of better outcomes in terms of death and complications compared with open surgery, especially in patients presenting with hemodynamic instability and in pregnant woman. Moreover, early and late reintervention and open conversion are still a considerable concern of emergent endovascular repair.

## Figures and Tables

**Table 1:** Baseline clinical characteristics at presentation.

	OSR (%)	EVT (%)	*p*-Value
Male sex	63/185 (34%)	57/165 (34.5%)	1
Female sex	68/185 (36.75%)	27/165 (16.5%)	0.01
Sex non reported	54/185 (29.25%)	81/165 (49%)	-
Mean age	41.5 ± 15.3	53.5 ± 16.9	0.02
SAPAs	23/185 (12.4%)	69/165 (41.8%)	<0.01
Hemodynamic stability	19 (10.6%)	81 (49.1%)	<0.01
Hemodynamic instability	142 (76.5%)	81 (49.1%)	<0.01
Hemodynamic status non reported	24 (12.9%)	3 (1.8%)	-
Rupture during pregnancy	79 (42.7%)	7 (4.2%)	0.01

OSR: open surgical repair, EVT: endovascular treatment, SAPA: splenic artery pseudoaneurysm.

**Table 2:** Peri-operative results.

	OSR	EVT	*p*-Value
Overall mortality	24/185 (12.9%)	13/165 (7.8%)	0.84
Mortality in HD stable patients	0/19 (0%)	7/81 (8.6%)	Logistic regression analysis on HD instability as predictor of mortality: *p*-value (OSR): 0.02 *p*-value (EVT): 0.18
Mortality in HD unstable patients	13/142 * (9.1%)	3/81 * (3.7%)	
Early reintervention conversion to OSR	5/185 (2.7%) -	37/165 (22.4%) 31/165 (18.8%)	<0.01 -
Reinterventions/conversions in HD stable patients	2/19 (10.5%)	9/81 (11.1%)	Logistic regression analysis on HD instability as predictor of reintervention: *p*-value (OSR): 0.30 *p*-value (EVT): 0.01
Reinterventions/conversions in HD stable patients	1/142 * (7%)	21/81 * (25.9%)	

OSR: open surgical repair, EVT: endovascular repair, HD: hemodynamics, * HD status non reported in 24 pts in the OSR group and in 3 pts in the EVT group.

**Table 3:** Peri-operative complications.

	OSR	EVT	Total	*p*-Value
Systemic complications	4/185 (2 sepsis, 1 stroke, 1 ileus)	4/165 (2 MI, 1 pneumonia, 1 ileus)	8/350	1
Splenic abscess	1/185	3/165	4/350	0.8
Splenic infarction	0/185	4/165	4/350	0.3
Pancreatitis	2/185	0/165	2/350	0.7
Total	7/185 (3.8%)	11/165 (6.7%)	18/350 (5.1%)	0.8

OSR: open surgical repair, EVT: endovascular repair, MI: myocardial infarction.

## References

[1] Chaer R.A., Abularrage C.J., Coleman D.M., Eslami M.H., Kashyap V.S., Rockman C., Murad M.H. (2020). The Society for Vascular Surgery clinical practice guidelines on the management of visceral aneurysms. J. Vasc. Surg..

[2] Björck M., Koelemay M., Acosta S., Goncalves F.B., Kölbel T., Kolkman J., Lees T., Lefevre J., Menyhei G., Oderich G. (2017). Editor’s Choice—Management of the Diseases of Mesenteric Arteries and Veins: Clinical Practice Guidelines of the European Society of Vascular Surgery (ESVS). Eur. J. Vasc. Endovasc. Surg..

[3] Cordova A.C., Sumpio B.E. (2013). Visceral Artery Aneurysms and Pseudoaneurysms—Should They All be Managed by Endovascular Techniques?. Ann. Vasc. Dis..

[4] Page M.J., McKenzie J.E., Bossuyt P.M., Boutron I., Hoffmann T.C., Mulrow C.D., Shamseer L., Tetzlaff J.M., Akl E.A., Brennan S.E. (2021). The PRISMA 2020 statement: An updated guideline for reporting systematic reviews. BMJ.

[5] Kramann B., Daoyu H., Kubale R., Schneider G. (1995). Erfahrungen mit der endovaskulären Embolisationstherapie von Aneurysmata der Splanchnikusarterien—Bericht über 13 Fälle. [Experiences with the endovascular embolization therapy of aneurysms of the splanchnic arteries—A report on 13 cases]. Rofo.

[6] Benz C.A., Jakob P., Jakobs R., Riemann J.F. (2000). Hemosuccus Pancreaticus—A Rare Cause of Gastrointestinal Bleeding: Diagnosis and Interventional Radiological Therapy. Endoscopy.

[7] Kim Y.S., Jung Y.H., Han K.W., Joo H.S., Cho Y.K., Park J.W., Lee S.H., Kim H.C., Park S.I., Jung I.K. (2004). A case of the ruptured splenic artery aneurysm treated with transcatheter embolization. Korean J. Gastroenterol..

[8] Hasaj O., Di Stasi C., Perri V., Tringali A., Costamagna G. (2004). Hemosuccus Pancreaticus Secondary to Intraductal Rupture of a Primary Splenic Artery Aneurysm: Diagnosis by ERCP and Successful Management by Interventional Radiology. Endoscopy.

[9] Kuzuya A., Mizuno K., Miyaka H., Iyomasa S., Matsuda M. (2006). Hemosuccus Pancreaticus Caused by Rupture of a True Splenic Artery Aneurysm following a Failure of Coil Embolization. Ann. Vasc. Surg..

[10] Laganà D., Carrafiello G., Mangini M., Dionigi G., Caronno R., Castelli P., Fugazzola C. (2006). Multimodal approach to endovascular treatment of visceral artery aneurysms and pseudoaneurysms. Eur. J. Radiol..

[11] Sachdev U., Baril D.T., Ellozy S.H., Lookstein R.A., Silverberg D., Jacobs T.S., Carroccio A., Teodorescu V.J., Marin M.L. (2006). Management of aneurysms involving branches of the celiac and superior mesenteric arteries: A comparison of surgical and endovascular therapy. J. Vasc. Surg..

[12] Fernández E.P., de la Cruz Burgos R., Del Cerro González J.V., Polo M.R. (2007). Rotura de bazo espontánea secundaria a aneurisma intraesplénico [Spontaneous rupture of the spleen secondary to intrasplenic aneurysm]. Radiologia.

[13] Tulsyan N., Kashyap V.S., Greenberg R.K., Sarac T.P., Clair D.G., Pierce G., Ouriel K. (2007). The endovascular management of visceral artery aneurysms and pseudoaneurysms. J. Vasc. Surg..

[14] Sadat U., Noor N., Tang T., Varty K. (2007). Emergency endovascular repair of ruptured visceral artery aneurysms. World J. Emerg. Surg..

[15] Loffroy R., Guiu B., Cercueil J.-P., Lepage C., Cheynel N., Steinmetz E., Ricolfi F., Krausé D. (2008). Transcatheter Arterial Embolization of Splenic Artery Aneurysms and Pseudoaneurysms: Short- and Long-Term Results. Ann. Vasc. Surg..

[16] Pasklinsky G., Gasparis A.P., Labropoulos N., Pagan J., Tassiopoulos A.K., Ferretti J., Ricotta J.J. (2008). Endovascular Covered Stenting for Visceral Artery Pseudoaneurysm Rupture: Report of 2 Cases and a Summary of the Disease Process and Treatment Options. Vasc. Endovasc. Surg..

[17] Yamamoto S., Hirota S., Maeda H., Achiwa S., Arai K., Kobayashi K., Nakao N. (2008). Transcatheter Coil Embolization of Splenic Artery Aneurysm. Cardiovasc. Interv. Radiol..

[18] Bjerring O.S., Pørneki J.C., Justesen P.E. (2008). Endovaskulaert behandlet rumperet aneurisme på arteria lienalis [Endovascular treatment of ruptured splenic artery aneurysm]. Ugeskr. Laeger.

[19] Zubaidi A. (2009). Rupture of multiple splenic artery aneurysms: A common presentation of a rare disease with a review of literature. Saudi J. Gastroenterol..

[20] Manian U., Badri H., Coyne P., Nice C., Ashour H., Bhattacharya V. (2009). Endovascular Treatment of a Ruptured Splenic Artery Aneurysm using Amplatzer^®^ Vascular Plug. Int. J. Biomed. Sci..

[21] Huang Y.-C., Xie Z.-Y., Tseng H.-S., Yang C.-F., Hsiao L.-T. (2009). Splenic artery pseudoaneurysm with rupture by transformed splenic marginal zone B cell lymphoma. Ann. Hematol..

[22] Iyanaga M., Watts S., Kasai T. (2010). A Patient with Splenic Artery Aneurysm Rupture and the Importance of Rapid Sonography in the ED. Emerg. Med. Int..

[23] Sadhu S., Sarkar S., Verma R., Dubey S., Roy M. (2010). Haemosuccus pancreaticus due to true splenic artery aneurysm: A rare cause of massive upper gastrointestinal bleeding. J. Surg. Case Rep..

[24] Ohyama Y., Ishida H., Yoshida C., Konno J., Hoshino T., Watanabe H., Kudoh Y., Furukawa K., Watanabe T. (2010). Pseudoaneurysm in a chronic pancreatitis patient: Report of a case, with emphasis on contrast-enhanced sonograms. J. Med. Ultrason..

[25] Naitoh I., Ando T., Shimohira M., Nakazawa T., Hayashi K., Okumura F., Miyabe K., Yoshida M., Togawa H., Sasaki S. (2010). Hemosuccus pancreaticus associated with segmental arterial mediolysis successfully treated by transarterial embolization. JOP.

[26] Eng C.W., Venkatesh S.K. (2010). Clinics in diagnostic imaging (131). Multiple visceral mycotic aneurysms. Singapore Med. J..

[27] Spiliopoulos S., Sabharwal T., Karnabatidis D., Brountzos E., Katsanos K., Krokidis M., Gkoutzios P., Siablis D., Adam A. (2012). Endovascular Treatment of Visceral Aneurysms and Pseudoaneurysms: Long-term Outcomes from a Multicenter European Study. Cardiovasc. Interv. Radiol..

[28] Cochennec F., Riga C., Allaire E., Cheshire N., Hamady M., Jenkins M., Kobeiter H., Wolfe J., Becquemin J., Gibbs R. (2011). Contemporary Management of Splanchnic and Renal Artery Aneurysms: Results of Endovascular Compared with Open Surgery from Two European Vascular Centers. Eur. J. Vasc. Endovasc. Surg..

[29] Gehlen J.M.L.G., Heeren P.A.M., Verhagen P.F., Peppelenbosch A.G. (2011). Visceral Artery Aneurysms. Vasc. Endovasc. Surg..

[30] Fankhauser G.T., Stone W.M., Naidu S.G., Oderich G.S., Ricotta J.J., Bjarnason H., Money S.R., Mayo Vascular Research Center Consortium (2011). The minimally invasive management of visceral artery aneurysms and pseudoaneurysms. J. Vasc. Surg..

[31] Etezadi V., Gandhi R.T., Benenati J.F., Rochon P., Gordon M., Benenati M.J., Alehashemi S., Katzen B.T., Geisbüsch P. (2011). Endovascular Treatment of Visceral and Renal Artery Aneurysms. J. Vasc. Interv. Radiol..

[32] Ferrero E., Ferri M., Viazzo A., Robaldo A., Carbonatto P., Pecchio A., Chiecchio A., Nessi F. (2011). Visceral Artery Aneurysms, an Experience on 32 Cases in a Single Center: Treatment from Surgery to Multilayer Stent. Ann. Vasc. Surg..

[33] Rosales-Zabal J.M., Navarro-Jarabo J.M., Rivera-Irigoin R., Perez-Aisa A., Marcos-Herrero M., Sanchez-Cantos A.M. (2011). Rupture of a splenic pseudoaneurysm in the colon as an unusual cause of rectal bleeding. Color. Dis..

[34] Wierzbicki T., Szmeja J., Borejsza-Wysocki M., Męczyński M., Smuszkiewicz P., Katulska K., Drews M. (2012). Massive bleeeding from upper gastrointestinal tract as a symptom of rupture of splenic artery aneurysm to stomach. Med. Sci. Monit..

[35] Taslakian B., Khalife M., Faraj W., Mukherji D., Haydar A. (2012). Pancreatitis-associated pseudoaneurysm of the splenic artery presenting as lower gastrointestinal bleeding: Treatment with transcatheter embolisation. BMJ Case Rep..

[36] Khurana J., Spinello I.M. (2012). Splenic artery aneurysm rupture: A rare but fatal cause for peripartum collapse. J. Intensive Care Med..

[37] Mazzaccaro D., Carmo M., Nano G., Barbetta I., Settembrini A.M., Occhiuto M.T., Stegher S., Dallatana R., Malacrida G., Settembrini P.G. (2015). Treatment options for visceral artery aneurysms: Ten year experience. J. Cardiovasc. Surg..

[38] Miao Y.-D., Ye B. (2013). Intragastric rupture of splenic artery aneurysms: Three case reports and literature review. Pak. J. Med. Sci..

[39] Amico E.C., Alves J.R. (2014). Rupture of splenic artery pseudoaneurysm. Pancreatology.

[40] Ünlü Ç., Heuvel D.A.v.D., Leeuwis J.W., de Vries J.-P.P. (2014). Ruptured Aneurysm of the Splenic Artery Associated with Fibromuscular Dysplasia. Ann. Vasc. Surg..

[41] Roberts K., McCulloch N., Forde C., Mahon B., Mangat K., Olliff S., Jones R. (2015). Emergency Treatment of Haemorrhaging Coeliac or Mesenteric Artery Aneurysms and Pseudoaneurysms in the Era of Endovascular Management. Eur. J. Vasc. Endovasc. Surg..

[42] Akbulut S., Otan E. (2015). Management of Giant Splenic Artery Aneurysm: Comprehensive Literature Review. Medicine.

[43] Schatz R.A., Schabel S., Rockey D.C. (2015). Idiopathic Splenic Artery Pseudoaneurysm Rupture as an Uncommon Cause of Hemorrhagic Shock. J. Investig. Med. High Impact Case Rep..

[44] Pitton M.B., Dappa E., Jungmann F., Kloeckner R., Schotten S., Wirth G.M., Mittler J., Lang H., Mildenberger P., Kreitner K.-F. (2015). Visceral artery aneurysms: Incidence, management, and outcome analysis in a tertiary care center over one decade. Eur. Radiol..

[45] Reed N.R., Oderich G.S., Manunga J., Duncan A., Misra S., de Souza L.R., Fleming M., de Martino R. (2015). Feasibility of endovascular repair of splenic artery aneurysms using stent grafts. J. Vasc. Surg..

[46] Won Y., Lee S., Kim Y., Ku Y. (2015). Clinical efficacy of transcatheter embolization of visceral artery pseudoaneurysms using N-butyl cyanoacrylate (NBCA). Diagn. Interv. Imaging.

[47] Mizuno S., Imai H., Takaki H., Tanemura A., Kuriyama N., Sakurai H., Yamakado K., Sakuma H., Isaji S. (2015). Spontaneous Rupture of an Intrasplenic Aneurysm After Pancreaticoduodenectomy for Pancreatic Ductal Adenocarcinoma. J. Emerg. Med..

[48] Matsuda Y., Sakamoto K., Nishino E., Kataoka N., Yamaguchi T., Tomita M., Kazi A., Shinozaki M., Makimoto S. (2015). Pancreatectomy and splenectomy for a splenic aneurysm associated with segmental arterial mediolysis. World J. Gastrointest. Surg..

[49] Figueroa-Jiménez L.A., González-Márquez A.L., Negrón-García L., Rosas-Soler F., Dones-Rodríguez A., De La Paz-López M., Santiago-Casiano M., Rodríguez-Cruz E., Cáceres-Pérkins W., Béez-Díaz L. (2015). Uncommon cause of life-threatening retroperitoneal hemorrhage in a healthy young Hispanic patient: Splenic artery aneurysm rupture. Bol. Asoc. Medica Puerto Rico.

[50] Guo B., Guo D., Xu X., Chen B., Shi Z., Luo J., Jiang J., Fu W. (2016). Early and intermediate results of endovascular treatment of symptomatic and asymptomatic visceral artery aneurysms. J. Vasc. Surg..

[51] Tannoury J., Honein K., Abboud B. (2016). Splenic artery aneurysm presenting as a submucosal gastric lesion: A case report. World J. Gastrointest. Endosc..

[52] Jiang R., Ding X., Jian W., Jiang J., Hu S., Zhang Z. (2016). Combined Endovascular Embolization and Open Surgery for Splenic Artery Aneurysm with Arteriovenous Fistula. Ann. Vasc. Surg..

[53] Davis T., Minardi J., Knight J., Larrabee H., Schaefer G. (2015). Ruptured Splenic Artery Aneurysm: Rare Cause of Shock Diagnosed with Bedside Ultrasound. West J. Emerg. Med..

[54] Aoki M., Hagiwara S., Miyazaki M., Kaneko M., Murata M., Nakajima J., Ohyama Y., Tamura J., Tsushima Y., Oshima K. (2016). Genuine splenic artery aneurysm rupture treated by N-butyl cyanoacrylate and metallic coils under resuscitative endovascular balloon occlusion of the aorta. Acute Med. Surg..

[55] Sul H.R., Lee H.W., Kim J.W., Cha S.J., Choi Y.S., Kim G.H., Kwak B.K. (2016). Endovascular management of hemosuccus pancreaticus, a rare case report of gastrointestinal bleeding. BMC Gastroenterol..

[56] Toukouki A., Verbeeck N., Weber J., Lens V. (2016). Intragastric Rupture of a Splenic Artery Aneurysm Associated with a Pancreatic Cancer. J. Belg. Soc. Radiol..

[57] Regus S., Lang W. (2016). Rupture Risk and Etiology of Visceral Artery Aneurysms and Pseudoaneurysms: A Single-Center Experience. Vasc. Endovasc. Surg..

[58] Frasnelli A. (2016). Successful resuscitation after splenic artery aneurysm rupture. J. Emerg. Trauma Shock.

[59] Quiles M.P., López V., Fernández J., Cascales P., Valero J.S., Parrilla P. (2016). Spontaneous rupture of a splenic aneurysm in classic Ehlers-Danlos syndrome. Cir. Esp..

[60] O’brien J., Muscara F., Farghal A., Shaikh I. (2016). Haematochezia from a Splenic Artery Pseudoaneurysm Communicating with Transverse Colon: A Case Report and Literature Review. Case Rep. Vasc. Med..

[61] Kingma K.D., van der Linden A.N., Roumen R.M.H. (2016). Rebleeding of a Splenic Artery Aneurysm after Coil Embolisation. Case Rep. Surg..

[62] Venturini M., Marra P., Colombo M., Panzeri M., Gusmini S., Sallemi C., Salvioni M., Lanza C., Agostini G., Balzano G. (2018). Endovascular Repair of 40 Visceral Artery Aneurysms and Pseudoaneurysms with the Viabahn Stent-Graft: Technical Aspects, Clinical Outcome and Mid-Term Patency. Cardiovasc. Interv. Radiol..

[63] Welch B.T., Brinjikji W., Stockland A.H., Lanzino G. (2017). Subarachnoid and intraperitoneal hemorrhage secondary to segmental arterial mediolysis: A case report and review of the literature. Interv. Neuroradiol..

[64] Blázquez M.J.P., Ortiz A.N. (2017). Hemosuccus pancreaticus secondary to pseudoaneurysm of the splenic artery. Rev. Esp. Enferm. Dig..

[65] Koutserimpas C., Papachristou E., Nikitakis N., Zannes N., Tellos A., Velimezis G. (2017). Spontaneous splenic artery aneurysm rupture in a 38-year old female: A case report. G. Chir..

[66] Meyer A., Uder M., Lang W., Croner R. (2010). Aneurysmen an viszeralen Arterien [Visceral artery aneurysms]. Zentralbl. Chir..

[67] Pfister K., Kasprzak P., Oikonomou K., Apfelbeck H., Derwich W., Uller W., Stehr A., Schierling W. (2018). Management von Viszeralarterienaneurysmen unter besonderer Berücksichtigung der Organperfusion—Erfahrungen über mehr als 20 Jahre [Management of Visceral Artery Aneurysms with Preservation of Organ Perfusion: More Than Twenty Years Experience]. Zentralbl. Chir..

[68] Hayashi S., Hosoda K., Nishimoto Y., Nonaka M., Higuchi S., Miki T., Negishi M. (2018). Unexpected intraabdominal hemorrhage due to segmental arterial mediolysis following subarachnoid hemorrhage: A case of ruptured intracranial and intraabdominal aneurysms. Surg. Neurol. Int..

[69] Junior P.R.P., Fagundes F.B., Marchon L.R.C., Maciel R.d.R.T., Martins I.M., Riguetti-Pinto C.R. (2018). Endovascular treatment of acute gastrointestinal bleeding from a large splenic artery pseudoaneurysm: Case report and literature review. J. Vasc. Bras..

[70] Ouchi T., Kato N., Nakajima K., Higashigawa T., Hashimoto T., Chino S., Sakuma H. (2018). Splenic Artery Aneurysm Treated with Endovascular Stent Grafting: A Case Report and Review of Literature. Vasc. Endovasc. Surg..

[71] Pratap A., Pokala B., Vargas L.M., Oleynikov D., Kothari V. (2018). Laparoscopic endoscopic combined surgery for removal of migrated coil after embolization of ruptured splenic artery aneurysm. J. Surg. Case Rep..

[72] Fang G., Chen B., Fu W., Guo D., Xu X., Jiang J., Luo J., Dong Z. (2018). Strategies for endovascular treatment of complicated splenic artery aneurysms. J. Vasc. Surg..

[73] Erben Y., Brownstein A.J., Rajaee S., Li Y., Rizzo J.A., Mojibian H., Ziganshin B.A., Elefteriades J.A. (2018). Natural history and management of splanchnic artery aneurysms in a single tertiary referral center. J. Vasc. Surg..

[74] Santos F.S., Sousa K.M.d.S., de Castro T.A.C., Neto F.C., de Oliveira R.G., de Araujo W.J.B., dos Santos L.C.P., de Souza R.C.A. (2018). Endovascular treatment of pseudoaneurysms secondary to chronic pancreatitis: Reports of two cases. J. Vasc. Bras..

[75] Nakamura T., Ikeda A., Itokawa Y., Kusumoto K., Nakai Y., Azechi H., Fujii S., Kusaka T., Kokuryu H. (2018). [A case of relapsed hemosuccus pancreaticus successfully treated with interventional radiology]. Nihon Shokakibyo Gakkai Zasshi.

[76] Olivieri J.F., Jeyakumar A., Shivaram G.M., Koo K.S., Monroe E.J. (2018). Emergent embolization of a ruptured splenic artery aneurysm complicating Menkes disease. Radiol. Case Rep..

[77] Martinelli O., Giglio A., Irace L., Di Girolamo A., Gossetti B., Gattuso R. (2019). Single-Center Experience in the Treatment of Visceral Artery Aneurysms. Ann. Vasc. Surg..

[78] Najafi A., Sheikh G.T., Binkert C. (2019). Extensive Embolization of Splanchnic Artery Aneurysms due to Segmental Arterial Mediolysis. Rofo.

[79] Patel R., Girgis M. (2019). Splenic artery pseudoaneurysm with hemosuccus pancreaticus requiring multimodal treatment. J. Vasc. Surg..

[80] Suri K., Tran K., Whang G. (2019). Gastric remnant perforation in a gastric bypass patient secondary to splenic artery pseudoaneurysm: Radiologic-surgical correlation. Clin. Imaging.

[81] Wang W., Chang H., Liu B., Wang W., Yu Z., Chen C., Li Y., Wang Z., Wang Y. (2020). Long-term outcomes of elective transcatheter dense coil embolization for splenic artery aneurysms: A two-center experience. J. Int. Med. Res..

[82] Nagao K., Kuroda K., Fujii M., Shirasaka D., Era Y., Tsuda K., Tanaka S., Miyazaki H., Hiemori A., Asada Y. (2020). Hematemesis due to rupture of splenic artery pseudoaneurysm in association with segmental arterial mediolysis: A case report. Nihon Shokakibyo Gakkai Zasshi.

[83] Effraemidou E., Souftas V., Kofina K., Karanikas M., Lyratzopoulos N. (2020). Spontaneous rupture of a splenic artery aneurysm treated with a spleen-preserving procedure: A case report. J. Surg. Case Rep..

[84] Hori E., Shibata T., Okamoto S., Kubo M., Horie Y., Kuroda S. (2020). An Intraabdominal Hemorrhage Affected by Catecholamine Surges Caused by Subarachnoid Hemorrhage:A Case Report. No Shinkei Geka.

[85] Chipaila J., Kato H., Iizawa Y., Motonori N., Noguchi D., Gyoten K., Hayasaki A., Fujii T., Tanemura A., Murata Y. (2020). Prolonged operating time is a significant perioperative risk factor for arterial pseudoaneurysm formation and patient death following hemorrhage after pancreaticoduodenectomy. Pancreatology.

[86] Kumari M., Parwez M., Jain A., Pandya B. (2020). Management of a delayed, post-traumatic rupture of splenic artery pseudoaneurysm in a patient with life threatening co-morbidities: A treatment challenge. Int. J. Surg. Case Rep..

[87] Tipaldi M.A., Krokidis M., Orgera G., Pignatelli M., Ronconi E., Laurino F., Laghi A., Rossi M. (2021). Endovascular management of giant visceral artery aneurysms. Sci. Rep..

[88] Carr S.C., Mahvi D.M., Hoch J.R., Archer C.W., Turnipseed W.D. (2001). Visceral artery aneurysm rupture. J. Vasc. Surg..

[89] Vanetta C., González Salazar E., Goransky J., Arbues G., Palavecino M. (2021). Tratamiento endovascular del aneurisma esplénico incidental y en la urgencia [Endovascular treatment of incidental and emergency splenic aneurysm]. Medicina.

[90] Sonanis S., Layton B., Nicholson O., Subar D. (2021). Splenic artery pseudoaneurysm and resultant haematosuccus pancreaticus. BMJ Case Rep..

[91] Law N.L., Villada A.F., Kruse M.J. (2021). Rupture of splenic artery aneurysm in a man with polycythemia vera and acquired von Willebrand syndrome. BMJ Case Rep..

[92] Borzelli A., Amodio F., Pane F., Coppola M., Silvestre M., Di Serafino M., Corvino F., Giurazza F., Niola R. (2021). Successful endovascular embolization of a giant splenic artery pseudoaneurysm secondary to a huge pancreatic pseudocyst with concomitant spleen invasion. Pol. J. Radiol..

[93] Xu Y., Wu Z. (2022). A case of a pregnant woman with a special splenic artery aneurysm. Malawi Med. J..

[94] Mulpuri V.B., Gurijala P., Yerolla B.R., Krishna R., Pandey A., Ramachandran G. (2022). Cross clamping of the supraceliac aorta is effective for bleeding control in ruptured giant splenic artery pseudoaneurysm when proximal and distal control of the splenic artery is not possible: A case report. J. Vasc. Bras..

[95] Tesolin D., Alaref A., Ibrahim M.F.K. (2021). A case of splenic artery aneurysm and rupture in a patient on a vascular endothelial growth factor inhibitor for renal cell carcinoma. Cancer Rep..

[96] Luan N.D., Duc N.M., Son N.H., Hien T.M., Huy L.A., Tai N.T., Kinh B.T., Loi H.M. (2021). A rare case report of acute upper gastrointestinal hemorrhage due to splenic artery pseudoaneurysm. SAGE Open Med. Case Rep..

[97] Vaughan E., Carlsson T., Brooks M., Elhodaiby M. (2022). Splenic artery aneurysm rupture in pregnancy: Challenges in diagnosis and the importance of multidisciplinary management. BMJ Case Rep..

[98] Patel D. (2022). Acute Abdomen from Spontaneous Splenic Artery Rupture with Coincidental Metastatic Disease: A Case Report. Am. J. Case Rep..

[99] Lee S.H., Yang S., Park I., Im Y.C., Kim G.Y. (2022). Ruptured splenic artery aneurysms in pregnancy and usefulness of endovascular treatment in selective patients: A case report and review of literature. World J. Clin. Cases.

[100] Okumura T., Kimura T., Nakajima D., Kondo S., Kako S., Takahashi Y., Yamaka K., Ichinohe F., Tsukahara Y., Nagaya T. (2022). Splenic artery pseudoaneurysm resulting from gastric ulcer presenting acute upper gastrointestinal bleeding. Radiol. Case Rep..

[101] Schellenberg M., Owattanapanich N., Emigh B., Nichols C., Dilday J., Ugarte C., Onogawa A., Matsushima K., Martin M.J., Inaba K. (2023). Pseudoaneurysms after high-grade blunt solid organ injury and the utility of delayed computed tomography angiography. Eur. J. Trauma Emerg. Surg..

[102] Pennetta F.F., Ferrer C., Tonidandel L., Coscarella C., Vagnarelli S., Giudice R. (2023). Disappearing multiple visceral aneurysms in Vascular Ehlers-Danlos syndrome. Vascular.

[103] Liu X., Chen S., Yang G., Hong J., Lin Y., Lin Z., Zhang Y., Chiang T.-Y. (2023). A super-selective coil impregnation therapy for pancreatic duct haemorrhage caused by pseudoaneurysm rupture. Technol. Health Care.

[104] Alexander E., Santos E. (2023). Endovascular management of incidentally discovered splenic arteriovenous fistula resulting from ruptured splenic aneurysm: Case report and review of the literature. Radiol. Case Rep..

[105] Aoki R., Kobayashi Y., Nawata S., Kamide H., Sekikawa Z., Utsunomiya D. (2023). Gastrointestinal Bleeding Due to the Rupture of Splenic Artery Caused by Pancreatic Carcinoma: A Case Requiring Repeated Transcatheter Arterial Embolization in a Short Period of Time. Interv. Radiol..

[106] Palughi M., Sirignano P., Stella N., Rossi M., Fiorani L., Taurino M. (2023). Rupture of Splenic Artery Aneurysm in Patient with ACTN2 Mutation. J. Clin. Med..

[107] LoCurto P., Farulla M.A., Di Lorenzo G., Amico M., Ciaccio G. (2019). Acute massive bleeding from splenic artery aneurysm rupture: A case report. G. Chir..

[108] Panzera F., Inchingolo R., Rizzi M., Biscaglia A., Schievenin M.G., Tallarico E., Pacifico G., Di Venere B. (2020). Giant splenic artery aneurysm presenting with massive upper gastrointestinal bleeding: A case report and review of literature. World J. Gastroenterol..

[109] Quandalle P., Gambiez L., Brami F., Ghisbain H., André J., Zahredine A., Saudemont A. (1998). Gastrointestinal hemorrhage caused by rupture of an aneurysm of visceral arteries. Presentation of 4 cases. Chirurgie.

[110] De Silva W.S.L., Gamlaksha D.S., Jayasekara D.P., Rajamanthri S.D. (2017). A splenic artery aneurysm presenting with multiple episodes of upper gastrointestinal bleeding: A case report. J. Med. Case Rep..

[111] Pinarbaşi B., Poturoğlu S., Yanar H., Güven K., Akyüz F., Dizdaroğlu F., Güllüoğlu M., Taviloğlu K., Kaymakoğlu S., Mungan Z. (2008). A rare cause of hemosuccus pancreaticus: Primary splenic artery aneurysm ruptured into pancreatic serous cystadenoma. Turk. J. Gastroenterol..

[112] Igari K., Ochiai T., Aihara A., Kumagai Y., Iida M., Yamazaki S. (2010). Hemosuccus pancreaticus caused by a primary splenic artery aneurysm as a rare cause of gastrointestinal bleeding: Report of a case. Int. Surg..

[113] Herrera-Fernández F.A., Palomeque-Jiménez A., Serrano-Puche F., Calzado-Baeza S.F., Reyes-Moreno M. (2014). Rupture of splenic artery pseudoaneurysm: An unusual cause of upper gastrointetinal bleeding. Cir. Cir..

[114] Carmeci C., McClenathan J. (2000). Visceral artery aneurysms as seen in a community hospital. Am. J. Surg..

[115] Popov P., Boskovic S., Sagic D., Radevic B., Ilijevski N., Nenezic D., Tasic N., Davidovic L., Radak D. (2007). Treatment of visceral artery aneurysms: Retrospective study of 35 cases. Vasa.

[116] Muscari F., Barret A., Chaufour X., Bossavy J., Bloom E., Pradère B., Gouzi J. (2002). Management of visceral artery aneurysms. Retrospective study of 23 cases. Ann. Chir..

[117] Rao S., Sivina M., Willis I., Sher T., Habibnejad S. (2007). Massive Lower Gastrointestinal Tract Bleeding Due to Splenic Artery Aneurysm: A Case Report. Ann. Vasc. Surg..

[118] Colombo P.L., Tinozzi F.P., Abelli M., Pini G., Benedetti M., Morone G., Moglia P., Albertario S., Laera M.R., Valenti L. (2002). Aneurysms of the visceral arteries: Report of 5 cases. Ann. Ital. Chir..

[119] Massani M., Bridda A., Caratozzolo E., Bonariol L., Antoniutti M., Bassi N. (2009). Hemosuccus pancreaticus due to primary splenic artery aneurysm: A diagnostic and therapeutic challenge. JOP.

[120] Hordiychuk A., Mehanna D. (2022). Spontaneous rupture of splenic artery pseudoaneurysm. J. Surg. Case Rep..

[121] Dave S.P., Reis E.D., Hossain A., Taub P.J., Kerstein M.D., Hollier L.H. (2000). Splenic Artery Aneurysm in the 1990s. Ann. Vasc. Surg..

[122] Windham T.C., Risin S.A., Tamm E.P. (2000). Spontaneous Rupture of a Nontraumatic Intrasplenic Aneurysm. N. Engl. J. Med..

[123] Gaglio P., Regenstein F., Slakey D., Cheng S., Takiff H., Rinker R., Dick D., Thung S. (2000). alpha-1 antitrypsin deficiency and splenic artery aneurysm rupture: An association?. Am. J. Gastroenterol..

[124] Asokan S., Chew E.K., Ng K.Y., Thanaletchimy N., Asmiati A., Kong N.M. (2000). Post partum splenic artery aneurysm rupture. J. Obstet. Gynaecol. Res..

[125] Arrieta F.M., Muguerza J., Sancho L.G., Ayuso M., Rustarazu M., Valenzuela P. (2000). Rupture of splenic artery aneurysm during pregnancy and posterior evolution of gestation. Zentralbl Gynakol..

[126] Shahabi S., Jani J., Masters L., Cobin L., Greindl J. (2000). Spontaneous Rupture of a Splenic Artery Aneurysm in Pregnancy: Report of Two Cases. Acta Chir. Belg..

[127] Sam C.E., Rabl M., Joura A.E. (2000). Aneurysm of the splenic artery: Rupture in pregnancy. Wien. Klin. Wochenschr..

[128] Fotopoulos N., Kyriakidis A., Dimopoulos K., Karkaletsis A., Faros E., Dalamarinis K., Raitsiou B., Antsaklis G. (2001). Splenic Artery Aneurysm Rupture. Dig. Surg..

[129] Balsarkar D.J., Joshi M.A. (2002). Rupture of splenic artery pseudoaneurysm presenting with massive upper gastrointestinal bleed. Am. J. Surg..

[130] Brocas E., Tenaillon A. (2002). Rupture spontanée de la rate au second trimestre de grossesse [Spontaneous splenic rupture in the second quarter of pregnancy]. Ann. Fr. Anesth. Reanim..

[131] Heestand G., Sher L., Lightfoote J., Palmer S., Mateo R., Singh G., Moser J., Selby R., Genyk Y., Jabbour N. (2003). Characteristics and Management of Splenic Artery Aneurysm in Liver Transplant Candidates and Recipients. Am. Surg..

[132] Selo-Ojeme D., Robarts P. (2004). Spontaneous rupture of splenic artery aneurysm in pregnancy. J. Obstet. Gynaecol..

[133] Woo E.Y., Fairman R.M. (2004). Treatment of multiple visceral aneurysms in a 20-year-old patient. J. Vasc. Surg..

[134] Khan H.R., Low S., Selinger M., Nelson N. (2004). Splenic artery aneurysm rupture in pregnancy. J. Coll. Physicians Surg. Pak..

[135] Popham P., Buettner A. (2003). Arterial aneurysms of the lienorenal axis during pregnancy. Int. J. Obstet. Anesth..

[136] Al Asfar F., Saber M., Dhar P.M., Al Awadhi N. (2004). Rupture of Splenic Artery Aneurysm during Labor: A Case Report of Maternal and Fetal Survival. Med. Princ. Pract..

[137] Dolar E., Uslusoy H., Kiyici M., Gurel S., Nak S.G., Gulten M., Zorluoglu A., Saricaoglu H., Memik F. (2005). Rupture of the splenic arterial aneurysm due to Behçet’s disease. Rheumatology.

[138] Saltzberg S.S., Maldonado T.S., Lamparello P.J., Cayne N.S., Nalbandian M.M., Rosen R.J., Jacobowitz G.R., Adelman M.A., Gagne P.J., Riles T.S. (2005). Is Endovascular Therapy the Preferred Treatment for All Visceral Artery Aneurysms?. Ann. Vasc. Surg..

[139] Brook O.R., Ghersin E., Guralnik L., Israelit S.H., Engel A. (2005). Abdominal apoplexy due to spontaneous rupture of an aberrant visceral artery pseudoaneurysm. Emerg. Radiol..

[140] Richardson A.J., Bahlool S., Knight J. (2006). Ruptured splenic artery aneurysm in pregnancy presenting in a manner similar to pulmonary embolus. Anaesthesia.

[141] Chaichian S., Mehdizadeh A., Akbarian A., Groohi B., Khanahmadi N., Alaghehbandan R. (2006). Rupture of Splenic Artery Aneurysm with Portal Hypertension During Pregnancy: A Case Report. J. Obstet. Gynaecol. Can..

[142] El-Shawarby S.A., Franklin O., South M., Goodman J. (2006). Caesarean splenectomy for spontaneous rupture of splenic artery aneurysm at 34 weeks gestation with survival of the mother and the preterm fetus. J. Obstet. Gynaecol..

[143] Cioppa T., De Stefano A., Marrelli D., Neri A., Rossi S., De Marco G., Pinto E., Roviello F. (2006). Pseudoaneurysm of the splenic artery fistulized in the stomach and associated to a pancreatic pseudocyst: Case report. Minerva Chir..

[144] Kalko Y., Ugurlucan M., Basaran M., Kafali E., Aydin U., Kafa U., Kosker T., Ozcaliskan O., Yilmaz E., Alpagut U. (2007). Visceral Artery Aneurysms. Heart Surg. Forum.

[145] Tarifa-Castila A., Salvoch-Arnedo F.J., Arín-Palacios B., Lera-Tricas J.M. (2007). Rupture of a splenic artery aneurysm. Cir. Esp..

[146] Mattick A., Gawthrope I. (2007). Splenic artery aneurysm rupture: Case report of this uncommon presentation. Emerg. Med. J..

[147] Toyoki Y., Hakamada K., Narumi S., Nara M., Ishido K., Sasaki M. (2008). Hemosuccus pancreaticus: Problems and pitfalls in diagnosis and treatment. World J. Gastroenterol..

[148] Upadhyaya P.K., Chava S., Bin-Sangheer S., Sudan R., Mittal S.K., Cemaj S. (2008). Delayed Rupture of a Splenic Artery Pseudoaneurysm After Biliopancreatic Diversion. Obes. Surg..

[149] Fernández E.L.-T., Delgado-Plasencia L., Arteaga-Gonzalez I., Carrillo-Pallares A., Diaz-Romero F. (2008). Posttraumatic Intrasplenic Pseudoaneurysm with High-Flow Arteriovenous Fistula: New Lessons to Learn. Eur. J. Trauma Emerg. Surg..

[150] Nordanstig J., Gerdes H., Kocys E. (2009). Spontaneous Isolated Dissection of the Celiac Trunk with Rupture of the Proximal Splenic Artery: A Case Report. Eur. J. Vasc. Endovasc. Surg..

[151] Sinha A., Meldrum D., Sinha B., Thakor A. (2009). Postpartum rupture of a splenic artery aneurysm presenting as disseminated intravascular coagulation. Int. J. Obstet. Anesth..

[152] Patrelli T.S., Anfuso S., Verrotti C., Fadda G.M., Gramellini D., Nardelli G.B. (2009). Intrapancreatic rupture of a splenic artery aneurysm during pregnancy—A rare case report with fetal and maternal survival. J. Matern. Neonatal Med..

[153] Kourabi M., Reibel N., Perez M., Grosdidier G. (2008). A serious late complication of non-operative management of splenic trauma: Rupture of splenic artery aneurysm. J. Chir..

[154] Mordant P., Trésallet C., Royer B., Brouquet A., Turrin N., Ménégaux F. (2008). Post-coital rupture of a splenic artery aneurysm. J. Chir..

[155] Mattick A., Gawthrope I. (2009). Splenic artery aneurysm rupture: Case report of this uncommon presentation. BMJ Case Rep..

[156] Sbihi L., Dafiri R. (2009). Unusual cause of hematemesis in a child: Rupture of a splenic artery aneurysm. J. Radiol..

[157] Varela C.A., Gómez J.M., Reina M.A., López A., Galindo S., Arruga A.M. (2009). Reversal of acenocoumarol anticoagulation with activated factor VII in massive hemorrhage following rupture of a splenic artery pseudoaneurysm. Rev. Esp. Anestesiol. Reanim..

[158] Chookun J., Bounes V., Ducassé J.L., Fourcade O. (2009). Rupture of splenic artery aneurysm during early pregnancy: A rare and catastrophic event. Am. J. Emerg. Med..

[159] Charokopos N.A., Foroulis C.N., Rouska E.G., Papakonstantinou C. (2009). Fatal rupture of splenic artery mycotic aneurysm after mitral valve replacement for infective endocarditis. Eur. J. Cardio-Thoracic Surg..

[160] Sarikaya S., Ekci B., Aktas C., Cetin A., Ay D., Demirag A. (2009). A rare clinic presentation of abdominal pain: Rupture of splenic artery aneurysm: A case report. Cases J..

[161] Thomson M.J., Seshadri S., Swami S., Strandvik G.F., Neales K. (2010). The pitfalls of protocols—A case of postpartum splenic artery aneurysm rupture. BMJ Case Rep..

[162] Ousadden A., Ibnmajdoub K.H., Elbouhaddouti H., Mazaz K., AitTaleb K. (2009). Intragastric rupture of a splenic artery aneurysm—A case report. Cases J..

[163] Betal D., Khangura J.S., Swan P.J., Mehmet V. (2009). Spontaneous ruptured splenic artery aneurysm: A case report. Cases J..

[164] Groussolles M., Merveille M., Alacoque X., Vayssiere C., Reme J.M., Parant O. (2010). Rupture of a Splenic Artery Aneurysm in the First Trimester of Pregnancy. J. Emerg. Med..

[165] Tsankova M., Jankova J., Dimitrova V., Grigorov G., Dzherov L. (2010). Rupture of splenic artery aneurysm--life-threatening condition for women during pregnancy and after birth (with report of one case). Akush Ginekol.

[166] Lakin R.O., Bena J.F., Sarac T.P., Shah S., Krajewski L.P., Srivastava S.D., Clair D.G., Kashyap V.S. (2011). The contemporary management of splenic artery aneurysms. J. Vasc. Surg..

[167] Rahmoune F.C., Aya G., Biard M., Belkhayat G., Hamza J., Leperc J., Ouchtati M. (2011). Splenic artery aneurysm rupture in late pregnancy: A case report and review of the literature. Ann. Fr. Anesth. Reanim..

[168] Dhinakar M., Al Mashini S., Golash V. (2011). Rupture of Splenic Artery Aneurysm during Pregnancy: A Report of two Cases. Oman Med. J..

[169] Michel P., Jarry J., Pagliano G. (2012). Faux anévrisme iatrogène de l’artère splénique après duodénopancréatectomie céphalique [Iatrogenic false aneurysm of the splenic artery after cephalic duodenopancreatectomy]. J. Mal. Vasc..

[170] Kalavský M., Smetka J. (2011). Krvácanie do hrubého creva spôsobené ruptúrou pseudoaneuryzmy arterie lienalis komplikujúcej pankreatickú pseudocystu [Large intestine bleeding caused by the lienal artery pseudoaneurysm rupture complicating a pancreatic pseudocyst]. Rozhl. Chir..

[171] Perino A., Proto E., Calagna G., Granese R., Agrusa A., Guarneri F., Cucinella G. (2012). Spontaneous rupture of splenic artery aneurysm in pregnancy: Is splenectomy always necessary?. Acta Obstet. Gynecol. Scand..

[172] Pavlis T., Seretis C., Gourgiotis S., Aravosita P., Mystakelli C., Aloizos S. (2012). Spontaneous Rupture of Splenic Artery Aneurysm during the First Trimester of Pregnancy: Report of an Extremely Rare Case and Review of the Literature. Case Rep. Obstet. Gynecol..

[173] Boumans D., Weerink L.B., Leyssius A.T.R., Swartbol P., Veneman T.F. (2013). Splenic Artery Rupture During Pregnancy Concealed by a Pancreatic Lymphangioma: A Rare Co-Occurrence. Ann. Vasc. Surg..

[174] Green A., Bowman-Burns C., Cumberbatch G. (2013). Abdominal pain and collapse in the emergency department. BMJ Case Rep..

[175] Boufettal H., Moussaïd I., Salmi S., Mahdaoui S., Hermas S., Samouh N. (2013). Spontaneous rupture of a splenic artery aneurysm in peri-partum. Ann. Fr. Anesth. Reanim..

[176] Benali M., Charrada H., Bouassida M., Bahloul A., Jmal K., Dhouib F., Saied M.R., Khaddar M.K. (2013). Splenic artery aneurysm rupture in late pregnancy: A case report. Ann. Fr. Anesth. Reanim..

[177] Oakley E., Ho J.D., Johnson V., VanCamp J., Melson T., Hick J.L. (2014). Splenic Artery Aneurysm: An Important Cause of Hemoperitoneum and Shock. J. Emerg. Med..

[178] Khoshnevis J., Lotfollahzadeh S., Sobhiyeh M.R., Sepas H.N., Nejad M.A., Rahbari A., Behnaz N., Mahdi Z. (2013). Ruptured Aneurysm of the Splenic Artery: A Rare Cause of Abdominal Pain after Blunt Trauma. Trauma Mon..

[179] Papadomichelakis A., Anyfantakis D., Kastanakis M., Karona P., Bobolakis E. (2014). Rupture of a splenic artery aneurysm in a previously healthy 53-year-old male. J. Med. Life.

[180] Phillips C., Bulmer J. (2013). Splenic Artery Aneurysm Rupture During Pregnancy. Nurs. Women’s Health.

[181] Goshayeshi L., Vosoghinia H., Rajabzadeh F., Ahadi M., Sakhmaresi T.A., Farzanehfar M.R. (2014). Splenic Artery Aneurysm as an Unusual Cause of New Onset Ascites: A Case Report. Middle East J. Dig. Dis..

[182] Jackson H.T., Diaconu S.C., Maluso P.J., Abell B., Lee J. (2014). Ruptured Splenic Artery Aneurysms and the Use of an Adapted Fast Protocol in Reproductive Age Women with Hemodynamic Collapse: Case Series. Case Rep. Emerg. Med..

[183] Kazaryan A.M., Wiborg J., Hauss K., Anundsen T.K., Flemmen O.J., Holm T.E., Lauzikas G. (2014). Spontaneous non-traumatic massive intraabdominal spleen bleeding in young females: Importance of ATLS principles and trauma alarm. Am. J. Case Rep..

[184] Zeren S., Bayhan Z., Sönmez Y., Mestan M., Korkmaz M., Kadıoglu E., Ucar B.I., Devir C., Ekici F.M., Sanal B. (2014). Spontaneous splenic artery aneurysm rupture: Mimicking acute myocardial infarct. Am. J. Emerg. Med..

[185] Abdulrahman A., Shabkah A., Hassanain M., Aljiffry M. (2014). Ruptured spontaneous splenic artery aneurysm: A case report and review of the literature. Int. J. Surg. Case Rep..

[186] Corey E.K., Harvey S.A., Sauvage L.M., Bohrer J.C. (2014). A Case of Ruptured Splenic Artery Aneurysm in Pregnancy. Case Rep. Obstet. Gynecol..

[187] Heitkamp A.C., Dickhoff C., Nederhoed J.H., Franschman G., de Vries J.I. (2015). Saved from a fatal flight: A ruptured splenic artery aneurysm in a pregnant woman. Int. J. Surg. Case Rep..

[188] Kataoka J., Nitta T., Fujii K., Yamaguchi T., Hirata Y., Kawakami K., Kawasaki H., Higashino T., Ishibashi T. (2015). A case of ruptured giant splenic artery aneurysm. Nihon Shokakibyo Gakkai Zasshi.

[189] Le Tinier B., Jungo-Nançoz C.J.-N., McCarey C., Jastrow N. (2015). Rupture of maternal splenic artery aneurysm and fetal demise. Clin. Exp. Obstet. Gynecol..

[190] Pejkic S., Tomic I., Opacic D., Pejinovic L., Grubor N., Cinara I., Davidovic L. (2015). Splenic artery aneurysms: Two cases of varied etiology, clinical presentation and treatment outcome. Srp. Arh. Celok. Lek..

[191] Sawicki M., Marlicz W., Czapla N., Łokaj M., Skoczylas M.M., Donotek M., Kołaczyk K. (2015). Massive Upper Gastrointestinal Bleeding from a Splenic Artery Pseudoaneurysm Caused by a Penetrating Gastric Ulcer: Case Report and Review of Literature. Pol. J. Radiol..

[192] Barišić T., Šutalo N., Letica L., Kordić A.V. (2015). Rupture of splenic artery aneurysm in primipara five days after cesarean section: Case report and review of the literature. Wien. Klin. Wochenschr..

[193] Chia C., Pandya G.J., Kamalesh A., Shelat V.G. (2015). Splenic Artery Pseudoaneurysm Masquerading as a Pancreatic Cyst—A Diagnostic Challenge. Int. Surg..

[194] Hiltrop N., Vanhauwaert A., Palmers P.-J.L.H., Cool M., DeBoever G., Lambrecht G. (2015). Hemosuccus pancreaticus caused by rupture of a splenic artery pseudoaneurysm complicating chronic alcoholic pancreatitis: An uncommon cause of gastrointestinal bleeding. Acta Gastro Enterol. Belg..

[195] Lee J.W., Kim T.N., Kim S.B., Kim K.H. (2016). Splenic rupture following transcatheter arterial embolization of splenic artery pseudoaneurysm caused by acute pancreatitis. Korean J. Intern. Med..

[196] Aydın M.T., Fersahoğlu M.M., Tezer S., Okuducu M., Ağca B., Memişoğlu K. (2016). Spontaneous rupture of the splenic artery aneurysm: A rare clinical presentation of acute abdomen. Ulus. Travma. Acil. Cerrahi. Derg..

[197] Robaldo A., Gramondo F., Beccaria F., Colotto P. (2016). Giant splenic artery aneurysm rupture. Clin. Case Rep..

[198] Monti J.D. (2016). A rare cause of abdominal pain and hypotension in pregnancy. J. Am. Acad. Physician Assist..

[199] Hostinská E., Huml K., Pilka R. (2016). Acute pancreatitis in pregnancy, complicated by rupture of aneurysm of artery lienalis. Ceska Gynekol..

[200] Jacobson J., Gorbatkin C., Good S., Sullivan S. (2017). Splenic artery aneurysm rupture in pregnancy. Am. J. Emerg. Med..

[201] Szpakowicz J., Szpakowicz P., Urbanik A., Markuszewski L. (2016). Splenic Artery Pseudoaneurysm Rupture into a Pancreatic Pseudocyst with its Subsequent Perforation as the Cause of a Massive Intra-Abdominal Bleeding—Case Report. Pol. Przegl. Chir..

[202] Kim J.H., Chung H.S., Kim J.H., Park S.Y., Lee S.B., Do B.S. (2017). Splenic artery aneurysm with the double-rupture phenomenon. Clin. Exp. Emerg. Med..

[203] Ologun G., Sharpton K., Granet P. (2017). Successful use of resuscitative endovascular balloon occlusion of the aorta in the treatment of ruptured 8.5-cm splenic artery aneurysm. J. Vasc. Surg..

[204] Bacalja I.D., Koprek D., Pavic P., Cvjetko I., Krpina K., Diklic D. (2017). Spontaneous rupture of a splenic artery aneurysm in a male patient. Neth. J. Med..

[205] Sakuraba S., Orita H., Ueda S., Tokuda S., Ito T., Kushida T., Sakurada M., Maekawa H., Wada R., Sato K. (2017). A Case of Segmental Arterial Mediolysis Presenting as Mucosal Gastric Hematoma. Case Rep. Gastrointest. Med..

[206] Maharaj R., Raghunanan B., Mohammed W., Rambally R., Sookdeo V.D., Harnanan D., Warner A.W. (2018). A rare case of massive lower gastrointestinal bleeding from a ruptured splenic artery aneurysm. J. Surg. Case Rep..

[207] Martin D., Farinha H.T., Dattner N., Rotman S., Demartines N., Sauvain O.M. (2018). Spontaneous non-traumatic splenic artery aneurysm rupture: A case report and review of the literature. Eur. Rev. Med. Pharmacol. Sci..

[208] Ktenidis K., Manaki V., Kapoulas K., Kourtellari E., Gionis M. (2018). Giant Splenic Aneurysm with Arteriovenous (A-V) Shunt, Portal Hypertension, and Ascites. Am. J. Case Rep..

[209] Chen G., Yang J., Qian G., Jiang K., Lv Y., Shi N., Zhu T. (2019). Spontaneous rupture of a splenic artery aneurysm with splenic epithelioid hemangioendothelioma: A case report. J. Int. Med. Res..

[210] Abdul R., Teelucksingh S., Omar M., Chow A.C., Boppana L.K.T., Goli S., Naraynsingh V., Teelucksingh S. (2019). Splenic artery pseudoaneurysm presenting with massive rectal bleeding. Radiol. Case Rep..

[211] Abhari P., Abhari S., Jackson A., Moustafa A.S.Z., Mercer L., Ashraf M. (2019). Splenic Artery Aneurysm Case Report. Case Rep. Obstet. Gynecol..

[212] Wiener Y., Tomashev R., Neeman O., Itzhakov Z., Heldenberg E., Melcer Y., Maymon R. (2019). Splenic artery aneurysms during pregnancy: An obstetric nightmare. Eur. J. Obstet. Gynecol. Reprod. Biol..

[213] Ballout A.R., Ghanem R., Nassar A., Hallal A.H., Ghulmiyyah L.M. (2019). Splenic Artery Aneurysm (SAA) Rupture in Pregnancy: A Case Report of a Rare but Life-Threatening Obstetrical Complication. J. Women’s Health Dev..

[214] Tlili A., Trigui A., Dkhil O., Feki W., Rejab H., Ben Ameur H., Boujelbene S., Mnif Z. (2019). Rupture d’un anévrisme de l’artère splénique en fin de grossesse: À propos d’un cas [Splenic artery aneurysm rupture at the end of pregnancy: A case study]. Pan Afr. Med. J..

[215] Pararas N., Rajendiran S., Taha I., Powar R.R., Holguera C., Tadros E. (2020). Spontaneous Rupture of a Huge Splenic Artery Aneurysm: A Case Report. Am. J. Case Rep..

[216] Montrief T., Parris M.A., Auerbach J.S., Scott J.M., Cabrera J. (2020). Spontaneous Splenic Artery Pseudoaneurysm Rupture Causing Hemorrhagic Shock. Cureus.

[217] Huff J., Valle O. (2020). Rupture of splenic artery aneurysm in pregnancy with double-rupture phenomenon: A case report. Case Rep. Women’s Health.

[218] Fujii M., Yamashita S., Fudono A., Yanai S., Tashiro J., Takenaka Y., Yamasaki K., Ito E., Masaki Y. (2020). Splenic artery aneurysm rupture during pregnancy: A case report of maternal and fetal survival. Int. J. Surg. Case Rep..

[219] Holt J.N., Schwalb E.H. (2020). A case of splenic artery pseudoaneurysm rupture presenting as rectal bleeding in a regional hospital. J. Surg. Case Rep..

[220] Vieujean S., Dauby M., Remacle G., Kridelka F., Dewandre P.Y., Capelle X. (2021). Spontaneous rupture of a splenic artery aneurysm during the third trimester of pregnancy. Rev. Med. Liege..

[221] Tan M.Y.Q., Wong A.J.T.-Y., Aung L., Ng W.M., Lee W.F., Lim B.L. (2021). Circulatory collapse from rupture of splenic artery aneurysm: A case study. Ann. Acad. Med. Singap..

[222] El Aidaoui K., Bensaad A., Habi J., El Yamani K., El Kettani C. (2021). Hemorrhagic Shock Revealing Rupture of Splenic Artery Pseudoaneurysm Three Years After Post-Traumatic Pancreatitis. Cureus.

[223] Wang A., Gao J. (2021). Spontaneous rupture of a splenic artery aneurysm during pregnancy. Asian J. Surg..

[224] Ornaghi S., Crippa I., Di Nicola S., Giardini V., La Milia L., Locatelli L., Corso R., Roncaglia N., Vergani P. (2022). Splenic artery aneurysm in obstetrical patients: A series of four cases with different clinical presentation and outcome. Int. J. Gynaecol. Obstet..

[225] Betancourth Alvarenga J.E., Santiago Martínez S., Jiménez Gómez S.J., San Vicente Vela M.B., Gaspar Pérez M., Álvarez García N., Güizzo J.R., Jiménez Arribas P., Esteva Miró C., Núñez García B. (2022). Management of splenic and/or hepatic pseudoaneurysm following abdominal trauma in pediatric patients. Cir. Pediatr..

[226] Chowdhury M.M., Quiyum M.A., Mohammed S., Karim R. (2022). Hemosuccus Pancreaticus: A Rare Cause of Gastrointestinal Bleeding. Mymensingh. Med. J..

[227] Yoshikawa C., Yamato I., Nakata Y., Nakagawa T., Inoue T., Nakatani M., Nezu D., Doi S., Kuroda Y., Fujii K. (2022). Giant splenic artery aneurysm rupture into the stomach that was successfully managed with emergency distal pancreatectomy. Surg. Case Rep..

[228] Hussein M.M., Al-Mollah M., Kanaan T. (2022). Splenic artery aneurysm rupture post-anterior cervical discectomy and fusion: Case report & literature review. Int. J. Surg. Case Rep..

[229] Hosseinzadeh A., Shahriarirad R., Majdazar V.A., Farsani M.M., Tadayon S.M.K. (2022). Spontaneous rupture of a large splenic artery aneurysm in a 59-year-old male patient with pemphigus vulgaris: A case report. J. Med. Case Rep..

[230] Hamilton E.J., Ngugi S., Kotakadeniya R. (2023). Surgical Management of Atraumatic Rupture of Splenic Artery Aneurysm with Spleen Preservation in a Regional Australian Hospital. Case Rep. Surg..

[231] Shalhub S., Nkansah R., El-Ghazali A., Hillenbrand C.J., Vaidya S.S., Schwarze U., Byers P.H. (2023). Splenic artery pathology presentation, operative interventions, and outcomes in 88 patients with vascular Ehlers-Danlos syndrome. J. Vasc. Surg..

[232] Barrionuevo P., Malas M.B., Nejim B., Haddad A., Morrow A., Ponce O., Hasan B., Seisa M., Chaer R., Murad M.H. (2019). A systematic review and meta-analysis of the management of visceral artery aneurysms. J. Vasc. Surg..

[233] Chiaradia M., Novelli L., Deux J.-F., Tacher V., Mayer J., You K., Djabbari M., Luciani A., Rahmouni A., Kobeiter H. (2015). Ruptured visceral artery aneurysms. Diagn. Interv. Imaging.

[234] Marone E.M., Mascia D., Kahlberg A., Brioschi C., Tshomba Y., Chiesa R. (2011). Is Open Repair Still the Gold Standard in Visceral Artery Aneurysm Management?. Ann. Vasc. Surg..

[235] Shera F.A., Shera T.A., Choh N.A., Bhat M.H., Shah O.A., Shaheen F.A., Robbani I., Gojwari T. (2023). Clinical Profile, Management, and Outcome of Visceral Artery Pseudoaneurysms: 5-Year Experience in a Tertiary Care Hospital. Int. J. Angiol..

[236] Gong C., Sun M.-S., Leng R., Ren H.-L., Zheng K., Wang S.-X., Zhu R.-M., Li C.-M. (2023). Endovascular embolization of visceral artery aneurysm: A retrospective study. Sci. Rep..

[237] Fargion A.T., Falso R., Speziali S., Biancofiore B., Esposito D., Giacomelli E., Dorigo W., Pulli R. (2023). Results of current endovascular treatments for visceral artery aneurysms. J. Vasc. Surg..

[238] Marone E.M., Rinaldi L.F. (2023). Current Debates in the Management of Visceral Artery Aneurysms: Where the Guidelines Collide. J. Clin. Med..

